# Upward Air Temperature Shifts and Acute Cardiovascular Events in Individuals with Atherosclerotic Cardiovascular Disease: A Time-Stratified Case-Crossover Study

**DOI:** 10.1016/j.lanepe.2026.101730

**Published:** 2026-06-04

**Authors:** Katharina Lechner, Siqi Zhang, Nils Krüger, Johannes Krefting, Kathrin Wolf, Marco Dallavalle, Tobias Dreischulte, Frank Offenborn, Fabian Starnecker, Moritz von Scheidt, Kai Chen, Annette Peters, Susanne Breitner, Alexandra Schneider, Heribert Schunkert

**Affiliations:** a Department of Cardiology, Deutsches Herzzentrum München, Universitätsklinikum der Technischen Universität München, Munich, Germany; b DZHK (German Centre for Cardiovascular Research), Partner site Munich, Munich Heart Alliance, Munich, Germany; c Helmholtz Zentrum München - German Research Center for Environmental Health (GmbH), Institute of Epidemiology, Munich, Germany; d Department of Environmental Health Sciences, Yale School of Public Health, New Haven, CT, United States of America; e Division of Pharmacoepidemiology and Pharmacoeconomics, Department of Medicine, Brigham and Women's Hospital and Harvard Medical School, Boston, MA, United States of America; f LMU Klinikum, Ludwig-Maximilians-Universität München, Institut für Allgemeinmedizin, Munich, Germany; g Allgemeine Ortskrankenkasse (AOK) Bayern, Munich, Germany; h Yale School of Public Health, Yale Center on Climate Change and Health, New Haven, CT, United States of America; i Faculty of Medicine, LMU Munich, Institute for Medical Information Processing, Biometry, and Epidemiology, Munich, Germany; j Harvard T.H. Chan School of Public Health, Department of Environmental Health, Boston, MA, United States of America

**Keywords:** Climate change, Upward temperature shifts, Ambient air temperature, Acute myocardial infarction, Coronary heart disease, Mortality

## Abstract

**Background:**

Global warming is an increasing health concern. While the health impact of heat has been widely studied, the effects of transient temperature fluctuations remain largely unknown. Here we evaluated the impact of upward air temperature shifts (uTS) on acute cardiovascular events and mortality in individuals with atherosclerotic cardiovascular disease (ASCVD).

**Methods:**

This time-stratified case-crossover study included 1,315,553 patients with ASCVD from a German health insurance between 2012 and 2020. Daily mean outside temperatures was estimated on a 1 × 1 km grid and, together with daily counts for acute coronary syndrome (ACS; n = 126,193), stroke (n = 154,122), hospitalizations for heart failure (n = 356,053), and all-cause mortality (n = 345,907), aggregated at the 3-digit postcode level. uTS was defined as days when mean air temperature exceeded the preceding seven-day average. Conditional Poisson regression was used to estimate acute and lagged associations, adjusting for time-varying confounders; area-specific estimates were pooled using multivariate meta-analysis.

**Findings:**

A 5 °C increase in uTS (observed on 185 days during the study period; 21 days/year), was associated with a higher risk for ACS (relative risk [RR] 1.03; 95% confidence interval [CI], 1.02–1.04), stroke (RR 1.02; 95% CI, 1.01–1.03), hospitalization for heart failure (RR 1.01; 95% CI, 1.01–1.02), and all-cause mortality (RR 1.03; 95% CI, 1.02–1.04). Associations were more pronounced during the warm season. The mean number of deaths attributable to uTS was 23 per 100,000 participants per year (95% CI, 18–29).

**Interpretation:**

Exposure to upward temperature shifts was associated with higher risks of ACS, stroke, hospitalizations for heart failure, as well as all-cause mortality in individuals with ASCVD. This was not fully explained by heat *per se*. Public health action is warranted, given the projected increase in temperature variability with climate change.

**Funding:**

This work was supported by a grant from the German Heart Foundation, grant no F/58/21 and the Bavarian State Ministry of Health and Care through the research project DigiMed Bayern (www.digimed-bayern.de), grant no DMB-1805-0001.

**Research in context**

**Evidence before this study**

Global warming is a growing health concern. Individuals with cardiovascular conditions are at heightened risk when exposed to environmental stressors. Recent research has addressed the health impact of heat, but the effects of temperature fluctuations on cardiovascular endpoints in very high-risk individuals are largely unknown. We conducted a PubMed search from database inception until May 15, 2025 using the search terms temperature variability, temperature shifts, cardiovascular events and mortality in English language. Our search revealed limited evidence regarding the association of upward temperature shifts on major adverse cardiovascular events in high-risk individuals. Therefore, we aimed to explore the associations between clinically meaningful upward temperature shifts and hospitalizations due to acute cardiovascular events and all-cause mortality in this susceptible population.

**Added value of this study**

Studying 1.3 million individuals with atherosclerotic cardiovascular diseases, we found that independent of heat per se, upward temperature shifts were associated with an increase of hospital admissions due to acute coronary syndrome and stroke on the day of the temperature increase, particularly in older adults. In terms of episodes per year, upward temperature shifts were more often observed than days with temperatures exceeding 30 °C. The overlap of the two phenomena is minimal, so that the effects we report here constitute a largely independent health threat for patients with atherosclerotic cardiovascular diseases. These findings shift the focus from static temperatures to dynamic temperature fluctuations, emphasizing the challenges of physiological adaptations to sudden environmental changes.

**Implications of all the available evidence**

Individuals living with atherosclerotic cardiovascular diseases are at heightened risk when exposed to environmental stressors. Temperature fluctuations, one facet of climate change, require significant physiological adaptations and may therefore be of particular concern for the large subgroup of the population living with cardiovascular conditions. While the relative risks conferred by upward temperature shifts may appear small at the individual level, they translate into a substantial burden at the population level, resulting in a high number of absolute events when considering the magnitude of exposure and the susceptibility of the affected population. These findings underscore the urgent need for practical approaches to climate change adaptation to protect vulnerable subgroups, particularly susceptible subgroups with cardiovascular disease.

## Introduction

Global warming due to climate change poses a major threat to human health in the 21st century.^1^ Consistent with this, non-optimal ambient temperatures have been identified as an important environmental risk factor for all-cause and cardiovascular mortality worldwide.^2^, ^3^, ^4^ Increasing temperature fluctuations, which require significant physiological adaptations, have been increasing due to anthropogenic warming.^5^

Atherosclerotic cardiovascular disease (ASCVD) remains the leading cause of morbidity and mortality worldwide.^2^ Individuals with established ASCVD appear particularly susceptible when exposed to environmental stressors.^6^^,^^7^ Epidemiologic studies of temperature extremes indicate increased cardiovascular risk in this population, potentially through acute physiological stress responses, highlighting the clinical relevance of temperature-related exposures.^8^, ^9^, ^10^, ^11^, ^12^

The association between upward temperature shifts (uTS) and the incidence of acute cardiovascular events and the risk of early death remain poorly characterized in individuals with ASCVD. Therefore, we examined the associations between uTS and these outcomes in a large ASCVD population in Germany. We hypothesized that uTS would be associated with an increase in acute cardiovascular events and higher all-cause mortality.

## Methods

### Study design

A time-stratified case-crossover design was used to evaluate associations between uTS and acute cardiovascular events. For each event, exposure on the event day was compared with exposure on control days from the same individual selected within the same year, month, and day of the week, thereby controlling for long-term time trends, seasonality, and day-of-week patterns. This within-person comparison inherently controls for time-invariant individual characteristics (e.g., chronic comorbidities and baseline medication use).^13^

### Study population

We utilized German insurance claims data from 1,315,553 publicly insured individuals aged 18 years and older diagnosed with ASCVD between January 2012 and December 2020. We identified our cohort based on routinely collected diagnostic codes. Eligible individuals were aged ≥18 years and had at least one inpatient or outpatient diagnosis between January 1, 2012, and December 31, 2020, indicating clinical manifestations of atherosclerotic or ischemic cardiovascular disease. Claims data was obtained from the German statutory health insurance Allgemeine Ortskrankenkasse (AOK) Bayern, which covers approximately 4,5 million individuals in Germany. No minimum number of years of insurance with AOK was needed. The data included de-identified demographic information on an individual level as well as all covered inpatient and outpatient encounters with the healthcare system, including ICD-10-GM codes, procedural codes, outpatient pharmacy-dispensed prescriptions, and mortality records. The prevalence of medication usage is shown in Table 1.

**Table 1:** Baseline characteristics of cases of patients.

Characteristics	Mean (SD) or N (%)
Demographics	
Age, mean (SD), year	75.6 (12.8)^a^
Female	353,4 (49.2)
Comorbidities	
Chronic ischemic heart disease	298,72 (41.6)
Peripheral artery disease	76,05 (10.6)
Carotid stenosis	73,77 (10.3)
Acute stroke	67,49 (9.4)
Chronic heart failure	209,76 (29.2)
Chronic kidney disease	179,75 (25.0)
Arterial hypertension	556,23 (77.5)
Diabetes mellitus	289,95 (40.4)
Dyslipidemia	367,43 (51.2)
Adiposity	149,20 (20.8)
Nicotine abuse	66,25 (9.2)
Medications	
Antiplatelet agents	128,41 (17.9)
ACE inhibitors	283,87 (39.5)
Angiotensin II receptor blockers	129,58 (18.1)
Calcium channel blockers	192,01 (26.7)
Beta blocking agents	329,67 (45.9)
Diuretics	377,34 (52.6)
Aldosterone antagonists	52,86 (7.4)
Insulins and analogs	76,25 (10.6)
Other antidiabetic agents	135,03 (18.8)

Data are expressed as N (%) unless otherwise specified. The number of cases of patients who died or were hospitalized due to acute coronary syndrome, heart failure, or stroke is 717,908.

ACE = angiotensin-converting enzyme, SD = standard deviation.

a Age at the first hospitalization due to acute coronary syndrome, heart failure, or stroke or at death.

### Exposure assessment

#### Air temperature

Mean air temperature reflects the overall ambient thermal level during the exposure window and is intended to capture the general background conditions participants experienced. We used daily mean air temperature (°C) values, estimated in grids of 1 × 1 km resolution covering Bavaria, southern Germany. We employed a multi-stage regression-based approach, integrating observed temperature data from weather stations, satellite-based land surface temperature, spatial predictors such as elevation and vegetation, and multiple land use predictors (e.g., percentages of urbanized areas, arable land, pastures, forests, and water bodies).^14^ The resulting models achieved strong performance (0.91 ≤ R^2^ ≤ 0.98 and 1.03 °C ≤ Root Mean Square Error (RMSE) ≤ 2.02 °C) across the state. Additionally, validation against a dense, independent monitoring network in Augsburg, Germany, further confirmed the models’ reliability (0.74 ≤ R^2^ ≤ 0.99, 0.87 °C ≤ RMSE ≤2.05 °C). Heat days were defined as days with maximum temperature ≥30 °C. They capture the frequency of extreme high-temperature events (above a predefined threshold of ≥30 °C) and are designed to represent episodic heat stress that may not be well represented by the mean. We calculated the temperature shifts by subtracting the preceding seven-day average from the mean air temperature of the day using the following formula:

$${TS}_{i}=T_{i}\;-\;{MA}_{7}\left(T_{i\;-\;1},T_{i\;-\;2},\ldots ,T_{i\;-\;7}\right)$$

where *TS*_*i*_ is the temperature shift for day *I*. *T*_*i*_ is the mean air temperature on day *I*, and *MA*_7_(*T*_*i* − 1_,*T*_*i* − 2_,…,*T*_*i* − 7_) is the seven-day moving average of temperature from the preceding seven days.

A positive temperature shift value represents an uTS. We chose a 5 °C increment for an upward temperature shift because it is close to one interquartile range (IQR) of the uTS exposure in our data. Using an increment near 1 IQR provides an effect estimate that reflects a typical, data-driven contrast and we considered a 5 °C increase in uTS to be of potential clinical relevance in patients with ASCVD. Upward temperature shifts quantify rapid warming relative to recent conditions, i.e., whether current temperatures are higher than those to which individuals were acclimatized in the immediately preceding week. Temperature variability reflects the degree of fluctuation in temperature over time and is intended to capture instability in exposure that may impose physiological strain beyond average conditions.

#### Relative humidity

We obtained daily mean relative humidity (RH, %) values, at a 1 km × 1 km spatial resolution across Bavaria, southern Germany, using a random forest modeling scheme that incorporated data from multiple ground- and satellite-based sources as well as previously modeled variables.^15^ The model exhibited high accuracy and low errors (R^2^ = 0.83 and RMSE = 5.07%) countrywide and in the external validation conducted in Augsburg, Germany (R^2^ ≥ 0.86, RMSE ≤5.45%).

#### Air pollution

We acquired daily mean air pollution (μg/m^3^) concentrations, i.e., particulate matter (PM) with an aerodynamic diameter equal to or smaller than 2.5 μm (PM_2.5_) or 10 μm (PM_10_), nitrogen dioxide (NO_2_) and ozone (O_3_), mapped in a spatial scale of approximately 2 km × 2 km across Germany by the German Environment Agency.^16^ These air pollution levels were predicted using an optimal interpolation technique, combining simulations from the chemistry-transport model REM-CALGRID with measurement data from the nationwide air monitoring network of the German federal states and the Federal Environment Agency.^16^ All models showed good performance (R^2^ ≥ 0.94, 0.71, 0.93 and RMSE ≤8, 23, 15 μg/m^3^ for PM_10_, NO_2_ and O_3_, respectively for the example year 2018).

#### Spatial linkage

This study averaged the daily exposures for 2012–2020 over 3-digit postcodes in Bavaria, southern Germany. Therefore, we aggregated the five-digit postcode polygons provided by the Federal Agency for Cartography and Geodesy (BKG) on behalf of Deutsche Post Direkt GmbH [© Deutsche Post Direkt GmbH (2019)] to 3-digit code areas. The dataset included 113 polygons. To compute the exposure estimates at the 3-digit postcode level, we intersected the exposure raster grids with the post code map. For each postcode, all centroids of the exposure raster cells falling within the boundary of the postal code were considered. We then linked the aggregated daily exposures to hospital admissions in Bavaria, via the postcode. The R spatial packages “sf” and “terra” (version 4.3.1) were used to compute the exposure estimates.

### Outcome assessment

All clinical outcomes were assessed in the inpatient setting as well as all-cause mortality. In concordance with the most frequently measured cardiovascular end points in clinical trials, we measured the following outcomes: ACS events were defined as an ICD-10-GM code for AMI (I21.x, I22.x) or unstable angina pectoris (I20.0) in combination with a procedural code for diagnostic cardiac catheterization (1–275.x), percutaneous coronary intervention (PCI) (8–837.x), or coronary artery bypass graft surgery (CABG) (5–36.x) within three days of the diagnosis, in concordance with previously conducted database studies that showed a high positive predictive value.^13^ AMI was defined accordingly. Stroke (I63.x, I64.x) and heart failure (I50.x) were defined as an ICD-10-GM code for the respective disease, as well as an inpatient hospitalization of at least one night. All-cause mortality was ascertained by the date of administrative death records in the insurance claims database, including death cases with and without prior hospitalizations. MACE was defined as a composite of AMI, stroke, and all-cause mortality, whichever occurred the earliest.

### Statistical analysis

We applied a two-stage statistical approach to assess the associations of daily mean temperature, heat days, and uTS with first hospital admissions due to AMI, ACS, heart failure, stroke, as well as all-cause mortality and MACE during 2012–2020, using separate models for each temperature metric. In the first stage, we conducted conditional quasi-Poisson regressions within each 3-digit postcode area (depicting the residence of individuals), allowing for overdispersion. The regression models were fitted with strata for year × month × day of the week (three-way interaction) to control for the area-specific time trend. By stratifying for month, the model adjusted for seasonal patterns in uTS and health outcomes, reducing potential seasonal confounding. This approach has been shown to be equivalent to the case-crossover study design using the conditional logistic regression model.^17^ For the daily mean temperature analysis, we employed a distributed lag non-linear model (DLNM), which incorporated a B-spline with three internal knots at the 10th, 75th, and 90th percentiles of the postcode-specific temperature distribution to represent the non-linear exposure-response function.^18^ The lag-response dimension was described by a natural cubic spline with an intercept and two internal knots placed at equally spaced values on the log scale for up to a lag of six days. We then reduced the exposure-lag-response association to the overall cumulative exposure-response association during the lag period. For the heat day analysis, we used DLNM with the same model specifications for the lag-response relationship as used in the daily mean temperature analysis. The exposure variable (heat day or not) was incorporated as a binary indicator. For the temperature shift analysis, we first checked the exposure-response relationship using DLNM, incorporating a natural spline of temperature shifts with two internal knots at the 10th and 75th percentiles of the postcode-specific temperature shift distribution. As we did not observe substantial deviation from linearity, temperature shift was included in the conditional Poisson regression model as a linear term in the main analysis. We assessed the immediate association using temperature shifts on the day of hospitalizations or deaths (lag 0 days), as well as potential delayed associations using temperature shifts one to six days preceding hospitalizations or deaths (lag 1 day – lag 6 days in separate single-lag models). The term lag day describes the difference in time between the day of the temperature shift and the occurrence of the outcomes, i.e., lag 0 refers to the outcomes occurring on the same day as the temperature shift, lag 1 refers to the outcomes occurring one day after the temperature shift, and so on. We excluded extreme temperature shift values above the 99th percentile or below the 1st percentile across the study period prior to model fitting as a robustness measure to reduce their potentially disproportionate influence in the conditional Poisson regression models. As a secondary analysis, we assessed the effect of temperature shift across different daily mean temperature ranges by including an interaction term between temperature shift, modeled as a linear term, and categorized daily mean temperature (below 0 °C, 0–10 °C, 10–20 °C, or above 20 °C) in the regression model, with adjustment for the categorized daily mean temperature.

In the second stage, we applied multivariate meta-analysis to derive the pooled associations of daily mean temperature, heat days, and temperature shifts with hospital admissions and mortality in Bavaria, Germany. Heat effects are presented as the relative risks (RR) with 95% confidence intervals (95% CIs) for an increase in the daily mean temperature from the 75th to the 97.5th percentile and cold effects as the RR (95% CI) for a decrease from the 25th to the 2.5th percentiles of the temperature distribution. The effects of temperature shifts are expressed as RR (95% CI) per 5 °C increment in uTS.

We further estimated the daily numbers of cause-specific hospital admissions and deaths attributable to uTS in each postcode area using the pooled effect estimates from the second stage through the following formula^19^:

$${AN}_{i,p}=N_{i,p}\times \left({RR}_{i,p}-1\right)÷{RR}_{i,p},with\;{RR}_{i,p}=\exp\left(\beta \times {uTS}_{i,p}\right)$$

where *AN*_*i*,*p*_ is the daily attributable number of hospital admissions/deaths in day *i* for postcode *p*; *N*_*i*,*p*_ is the daily total number of hospital admissions/deaths in day *i* for postcode *p*; *β* is the pooled coefficient per 1 °C increment in uTS derived from the second stage; *uTS*_*i*,*p*_ is the uTS in day *i* for postcode *p*. We then summed the daily AN over the study period and postcodes to get the total AN and calculated the population attributable risk, defined as AN per 100,000 participants per year. The lower and upper values of the 95% CI of the pooled coefficient are used to calculate the 95% CIs of AN using the above formula.

To explore potential effect modification by age and sex, we conducted stratified analyses for age in three categories (<65 years, 65–85 years and >85 years) and for sex (males and females). We also examined associations among individuals with complex multimorbidity,^20^ defined as being diagnosed with at least six diseases listed in Table S1 within 365 days prior to the index hospitalization or death. Additionally, we restricted the analyses to the warm (May to September) and cold (November to March) seasons to examine the seasonal variations. The statistical significance of the between-group differences in effect estimates was assessed by the two-sided Z-test.^21^

The robustness of the effects in temperature shifts was assessed by further adjustment for various exposures, including (1) daily mean temperature as a natural spline with three degrees of freedom (DoF) to account for potential non-linear relationships, (2) preceding 7-day average temperature as a natural spline with three DoF, (3) RH as a natural spline with three DoF, and (4) PM_10_, PM_2.5_, NO_2_, and O_3_ individually as a linear term. All additionally adjusted exposures were included at the same lag as temperature shifts to ensure consistency in temporal alignment.

All statistical analyses were performed using the R software (version 4.2.1) with the “gnm”, “dlnm”, and “mixmeta” packages. Statistical significance was determined based on a two-sided *p*-value threshold of less than 0.05.

### Role of the funding source

The funder had no role in the study design, data collection, data analysis, data interpretation, writing of the report, or the decision to submit the article for publication.

This study was approved by the Ethics Committee of the Technical University Munich under approval number [273/18S] on 27th June 2018 and [50/19S-SR] on 29th July 2019. All procedures were conducted in accordance with the applicable ethical standards and institutional requirements. Signed informed consent was waived due to the design of the study as a real-world evidence study based on routinely collected insurance claims data. The analysis was conducted using anonymized data, with no direct contact with study participants and no intervention.

## Results

### Exposure data and study population

The mean (SD) age in the population with pre-existing ASCVD was 76 (13) years, with 353,397 (49%) female participants (Table 1). Common comorbidities were arterial hypertension (556,238; 78%), dyslipidemia (367,428; 51%), diabetes mellitus (289,974; 40%), and chronic kidney disease (179,748; 25%).

We recorded 126,193 hospital admissions due to ACS (of which 83,895 cases were AMI), 154,122 due to stroke, 356,053 due to heart failure, and 345,907 deaths over nine years from January 1, 2012, to December 31, 2020, described in Table 2.

**Table 2:** Numbers of first hospital admissions due to acute coronary syndrome (ACS), acute myocardial infarction (AMI), heart failure (HF), and stroke, as well as all-cause deaths and first major adverse cardiovascular events (MACE)^a^ during the study period (2012–2020) in Bavaria.

Group	ACS	AMI	HF	Stroke	Death	MACE
Total	126,19	83,90	356,05	154,12	345,91	485,40
Age<65yrs	44,84	30,11	50,26	34,21	24,42	80,31
Age 65–85yrs	73,48	47,59	227,09	91,61	183,54	266,20
Age>85yrs	7,87	6,20	78,71	28,30	137,95	138,89
Males	80,48	54,75	175,36	73,38	166,26	245,39
Females	45,71	29,14	180,70	80,74	179,65	240,02
Multimorbid^b^	15,87	9,89	59,10	14,57	105,75	99,17
Warm season^c^	50,12	33,53	139,05	63,24	132,32	190,95
Cold season^c^	54,80	36,16	158,43	64,94	156,03	213,17

a Major adverse cardiovascular events (MACE) include deaths and hospital admissions due to MI and stroke.

b Complex multimorbidity was defined as having ≥ six diseases listed in Table S1 within 365 days prior to the index hospitalization or death.

c Warm season: May–September; Cold season: November–March.

The daily mean (SD) temperature over the study period was 9.2 °C (7.4 °C). Pearson correlation coefficients between temperature and other exposure metrics were generally weak to moderate. The average number of heat days (defined as days with air temperature ≥30 °C) per year was 10.

The daily mean uTS (i.e., the mean of all upward temperature shifts in the study period) was 2.4 °C, and the daily median uTS was recorded at 2.07 °C. Over the 9-year observational period, the average number of days with any uTS was 181 per year (range 161–204) across all postcode areas. Of these 181 days, an average of 9.4 days had a daily maximum temperature ≥30 °C. For the outcome assessment, an uTS of 5 °C (i.e., an increment of 5 °C as compared to the average of the 6 preceding days) was used, which was between the 75th (3.49 °C) and the 99th (6.94 °C) percentile of all observed uTS, as described in Table 3. The average number of days with uTS >5 °C across all postcode areas was 185 days over the study period (21 days per year on average). Of the 185 days with uTS >5 °C during the study period, an average of 29.3 days had a daily maximum temperature ≥30 °C.

**Table 3:** Description of area-specific daily exposure metrics during the study period (2012–2020).

Exposure	Mean	SD	Min	P1	P25	Median	P75	P99	Max
Daily mean temperature (°C)	9.2	7.4	−18.7	−6.7	3.4	9.2	15.0	24.1	30.3
Upward temperature shift (°C)	2.40	1.73	0.00	0.04	1.00	2.07	3.49	6.94	7.43
Number of days with uTS >5 °C	185	19	134	150	172	184	195	230	232
Number of heat days	90	34	3	10	72	88	107	158	168
Relative humidity (%)	79.0	11.5	33.6	50.6	70.6	80.8	88.5	96.8	99.5
PM_10_ (μg/m^3^)	13.1	8.5	0.5	2.4	7.6	11.3	16.4	44.0	275.9
PM_2.5_ (μg/m^3^)	10.0	7.2	0.5	1.6	5.5	8.3	12.4	38.2	191.6
NO_2_ (μg/m^3^)	12.2	8.3	0.5	2.8	6.4	9.9	15.5	42.4	97.2
O_3_ (μg/m^3^)	51.1	20.8	0	7.1	36.1	52.7	66.0	96.0	143.8

Max = maximum, Min = minimum, NO_2_ = nitrogen dioxide, O_3_ = ozone, PM_2.5_ = particulate matter with an aerodynamic diameter of ≤2.5 μm, PM_10_ = particulate matter with an aerodynamic diameter of ≤10 μm, SD = standard deviation, uTS = upward temperature shift, heat days where defined as days with air temperature >30 °C.

### Effects of mean ambient air temperatures and temperature variability on outcomes

#### Daily mean temperatures and heat days

The association between daily mean temperature and all-cause mortality or MACE was J-shaped, as shown in Figure S1. Across the spectrum, the lowest all-cause mortality rate was observed at 16.0 °C. By contrast, we did not observe significant associations between daily mean temperature and acute cardiovascular events.

The increased risk for all-cause mortality associated with high air temperatures was stronger in patients aged 65 years and older whereas it was consistent across biological sex, as shown in Table 4. Heat days were associated with higher all-cause mortality but showed no association with hospital admissions due to ASC, AMI, stroke or heart failure (data not shown).

**Table 4:** Pooled relative risks (with 95% confidence interval) for first hospital admissions due to acute coronary syndrome, acute myocardial infarction, heart failure, and stroke, as well as for all-cause mortality and first major adverse cardiovascular events^a^ associated with high air temperatures.

Analysis	ACS	AMI	HF	Stroke	Mortality	MACE
Main	0.93 (0.88, 1.00)	0.94 (0.87, 1.02)	1.01 (0.98, 1.05)	1.01 (0.95, 1.07)	1.14 (1.10, 1.18)^∗^	1.07 (1.03, 1.10)^∗^
Age <65yrs	1.01 (0.91, 1.12)	1.01 (0.90, 1.14)	0.98 (0.88, 1.09)	1.02 (0.90, 1.16)	1.08 (0.95, 1.23)	1.03 (0.96, 1.11)
Age ≥65yrs	0.89 (0.83, 0.96)^∗^	0.88 (0.80, 0.97)^∗^	1.01 (0.98, 1.05)	0.99 (0.94, 1.05)	1.14 (1.10, 1.19)^∗^	1.07 (1.03, 1.10)^∗^
Males	0.99 (0.93, 1.07)	0.98 (0.90, 1.07)	0.98 (0.93, 1.03)	1.00 (0.93, 1.07)	1.16 (1.09, 1.25)^∗^	1.09 (1.04, 1.14)^∗^
Females	0.86 (0.77, 0.95)^∗^	0.85 (0.74, 0.98)^∗^	1.04 (0.99, 1.10)	0.99 (0.93, 1.06)	1.11 (1.06, 1.17)^∗^	1.04 (1.00, 1.09)^∗^
Multimorbid^b^	0.92 (0.78, 1.10)	0.89 (0.70, 1.13)	1.02 (0.94, 1.12)	0.95 (0.79, 1.13)	1.21 (1.13, 1.29)^∗^	1.14 (1.07, 1.21)^∗^
Warm season^c^	0.95 (0.87, 1.04)	0.91 (0.80, 1.03)	1.00 (0.96, 1.04)	0.98 (0.91, 1.05)	1.18 (1.13, 1.23)^∗^	1.09 (1.05, 1.13)^∗^
Cold season^c^	0.95 (0.90, 1.02)	0.91 (0.84, 1.00)^∗^	0.97 (0.94, 1.01)	0.98 (0.92, 1.04)	0.97 (0.94, 1.01)	0.97 (0.94, 1.00)

Cumulative relative risks of daily mean temperature were derived from conditional Poisson regressions for lag 0–6 days in the main analysis. High temperature effects were estimated for increases in air temperature from the 75th to the 97.5th percentiles of temperature distributions.

ACS = acute coronary syndrome, AMI = acute myocardial infarction, HF = heart failure, MACE = major adverse cardiovascular events.

∗p < 0.05.

a Major adverse cardiovascular events include deaths and hospital admissions due to MI and stroke.

b Complex multimorbidity was defined as having ≥ six diseases listed in Table S1 within 365 days prior to the index hospitalization or death.

c Warm season: May–September; Cold season: November–March. Temperature effects were estimated for decreases/increases in air temperature based on season-specific temperature distributions.

#### Temperature variability

**Fig. 1:**
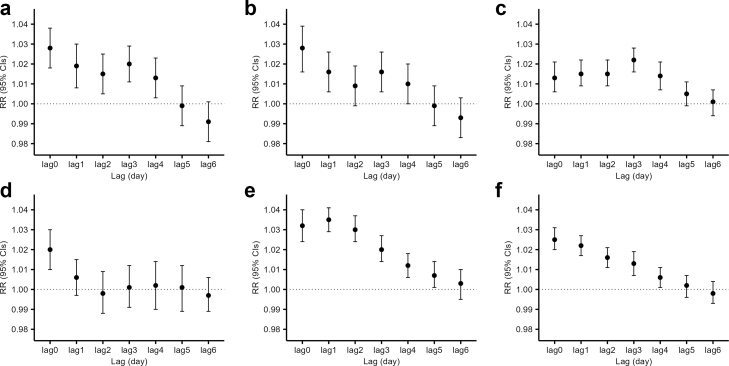
**Pooled relative risks (with 95% confidence interval) for first hospital admissions due to a) acute coronary syndrome, b) acute myocardial infarction, c) heart failure, and d) stroke, as well as for e) all-cause mortality and f) first major adverse cardiovascular events associated with per 5 °C greater upward temperature shift (uTS), by lag days.** Lag day 0 indicates the day when the uTS was observed. Conditional Poisson regression with a linear term of temperature variability was conducted for each postcode area. Postcode-specific estimates were pooled by random-effects meta-analysis.

As shown in Fig. 1, hospitalizations for AMI, ACS, stroke, and HF hospital admissions and all-cause mortality (as well as MACE rates) significantly increased at lag day 0, i.e., the day when the temperature shifted upward as compared to the average temperature of the seven days before. While the risk of stroke was only significantly elevated on this specific day, AMI, ACS, HF and mortality remained elevated up to five days or even increased. Specifically, the effects of upward temperature shifts on all-cause mortality and HF hospital admissions peaked at lag days 1 and 3, respectively.

The effects of upward temperature shifts broadly remained stable for daily mean temperature ranges between 0 °C and 20 °C, with the exception that in the highest range, i.e., temperatures over 20 °C, the effects on AMI, mortality and MACE became null after adjustment for confounders including average temperature on that day (Table 5).

**Table 5:** Pooled relative risks (with 95% confidence interval) for first hospital admissions due to acute coronary syndrome, acute myocardial infarction, heart failure, and stroke, as well as for all-cause mortality and first major adverse cardiovascular events associated with per 5 °C greater upward temperature shift in different ranges of daily mean temperature.

Outcome	Temp <0 °C	0 °C ≤ Temp <10 °C	10 °C ≤ Temp <20 °C	Temp ≥20 °C
ACS	1.02 (0.99, 1.05)	1.03 (1.02, 1.05)	1.03 (1.01, 1.05)	1.01 (0.96, 1.06)
AMI	1.02 (0.98, 1.07)	1.02 (1.00, 1.05)	1.04 (1.02, 1.06)	1.00 (0.92, 1.08)
HF	1.02 (1.00, 1.05)	1.02 (1.00, 1.03)	1.01 (1.00, 1.03)	1.00 (0.97, 1.04)
Stroke	1.04 (1.00, 1.09)	1.01 (0.99, 1.02)	1.03 (1.01, 1.05)	1.01 (0.96, 1.07)
Mortality	1.01 (0.99, 1.04)	1.03 (1.02, 1.05)	1.04 (1.03, 1.05)	0.99 (0.95, 1.03)
MACE	1.02 (0.99, 1.05)	1.02 (1.01, 1.03)	1.04 (1.03, 1.05)	0.99 (0.96, 1.02)

Conditional Poisson regression with an interaction term between temperature variability (linear term) and categorized daily mean temperature was conducted for each postcode area. Postcode-specific estimates were pooled by random-effects meta-analysis.

ACS = acute coronary syndrome, AMI = acute myocardial infarction, HF = heart failure, MACE = major adverse cardiovascular events.

At lag 0, while AMI and ACS hospital admissions reached a plateau at the 95th percentile of the temperature variability distribution, there is no such plateau for other outcomes, as shown in the exposure-response functions (Figure S2). Hospital admissions due to HF, stroke, as well as all-cause mortality showed a continuous increase with increasing uTS, even at the extremes of TV distribution.

In this sample, the average number of deaths attributable to uTS was 23 per 100,000 participants per year (95% CI, 18–29). Table S2 shows population attributable risk for cause-specific hospital admissions.

### Effect modifications by demographic and lifestyle factors, comorbidities, and ambient pollutants

**Fig. 2:**
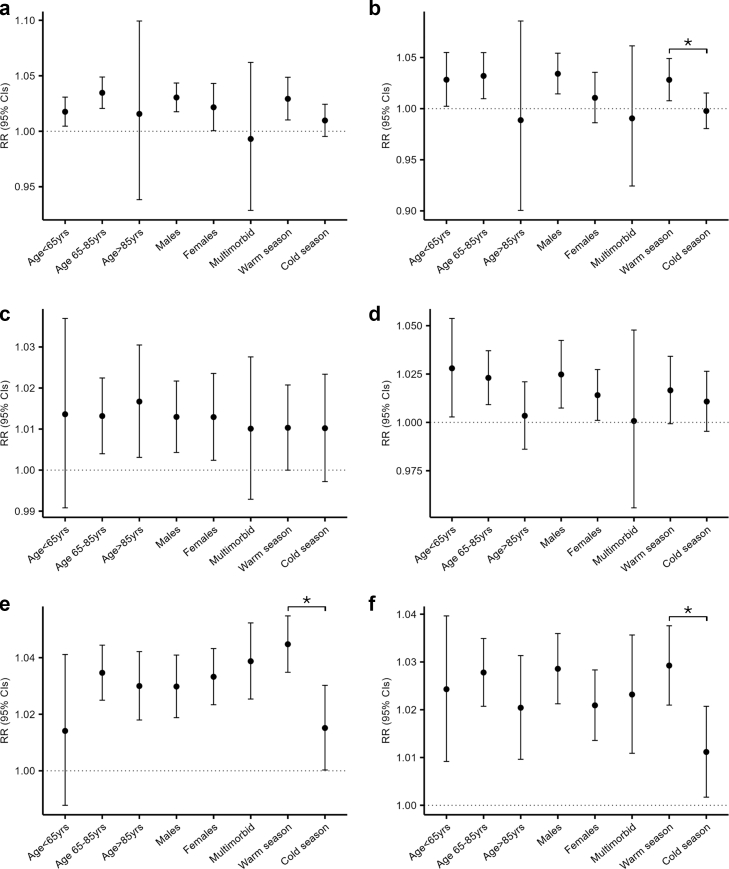
**Pooled relative risks (with 95% confidence interval) for first hospital admissions due to a) acute coronary syndrome, b) acute myocardial infarction, c) heart failure, and d) stroke, as well as for e) all-cause mortality and f) first major adverse cardiovascular events associated with per 5 °C greater upward temperature shift in different subgroups at lag 0 day.** ∗p value for the between-group difference <0.05. Conditional Poisson regression with a linear term of temperature variability was conducted for each postcode area. Postcode-specific estimates were pooled by random-effects meta-analysis.

The associations between outcomes and upward temperature shift at lag day 0 were broadly consistent across different age subgroups but were slightly stronger in male compared to female patients for AMI, stroke, and MACE, as shown in Table S3. Of note, the associations of AMI, mortality, and MACE were significantly stronger in the warm season than in the cold season (Fig. 2). For ACS and AMI, the associations with uTS were not significant in the cold season. In subgroups with markedly lower numbers of participants, non-significant associations were observed in the age <65 years group for all-cause mortality and heart failure, the age >85 years group for AMI, ACS and stroke, as well as in individuals with multimorbidity for AMI, ACS, heart failure, and stroke.

### Sensitivity analyses

At lag day 0, while the exposure-response function for AMI and ACS hospital admissions reached a plateau at the 95th percentile of the temperature variability distribution, there was no such plateau for the other outcomes studied. Hospital admissions due to heart failure, stroke, as well as all-cause mortality showed a continuous increase with increasing upward temperature shifts, even at the extremes of temperature variability distribution (data not shown).

Table S4 shows that even after adjusting for other exposure metrics at lag 0 day, the pooled RR for outcomes associated with 5 °C upward temperature shifts remained consistent with the associations observed in the main analysis.

Correlations between uTS and air pollutants were very small (Pearson correlation coefficient for uTS and PM_10_ = 0.09, PM_2.5_ = 0.04, NO_2_ = −0.01, O_3_ = 0.20), suggesting that strong confounding by air pollutants is unlikely (Table S5).

## Discussion

In this time-stratified case-crossover study of 1,315,553 individuals with ASCVD followed for up to 9 years, uTS were associated with acute cardiovascular events, including ACS, stroke, and hospitalization for heart failure, as well as all-cause mortality. Associations were more pronounced in the warm season and were observed at short lag times, with outcome-specific temporal patterns (e.g., stroke predominantly on the day of the temperature shift, whereas hospitalization for heart failure and mortality showed delayed peaks).

Importantly, uTS occurred more frequently than heat days (≥30 °C), and their overlap was limited. This indicates that short-term temperature changes constitute an exposure distinct from heat extremes and may contribute to the cardiovascular burden even on days not classified as extreme heat. Consistent with this, uTS-related risks were broadly present across a wide range of daily mean temperatures. In the highest mean temperature range (≥20 °C), associations for some outcomes attenuated after adjustment for temperature on the same day, suggesting that effects of heat per se may partially overlap with effects of temperature shifts under warm conditions.

Although the observed relative risks were modest, the public health relevance may be substantial given the high frequency of uTS and the vulnerability of the underlying population. We estimated that uTS were associated with an average of 23 deaths per 100,000 participants per year, underscoring the potential population-level impact of short-term temperature fluctuations in individuals with ASCVD.

With both low and high ambient air temperatures contributing to cardiovascular morbidity and mortality, prior studies have reported a higher ACS burden at low rather than high temperatures.^2^ In line with this, we observed no relevant association between high ambient temperatures and ACS-related hospital admissions. At the same time, rising global temperatures are expected to increase weather extremes and may also increase temperature variability.^5^

Over longer time horizons, heat may become a more important threat to cardiovascular health than cold. Supporting this hypothesis, we previously observed a non-significant decline in cold-related AMI risks over 28 years in the Augsburg KORA AMI registry, whereas heat-related AMI relative risk increased, suggesting a potential shift in the balance of temperature-related cardiovascular risk over time.^22^

Notably, our findings suggest that dynamic changes, i.e., uTS, represent a risk dimension that is distinct from heat days, and therefore warrant consideration alongside conventional metrics of heat exposure.

Our results are consistent with emerging literature linking temperature variability to acute cardiovascular events and mortality. A study from New York City reported that hour-to-hour temperature changes were positively associated with AMI hospitalizations.^23^ In Chinese cities, a 1 °C increase in temperature variability was associated with higher total mortality.^24^ Heterogeneity in how “temperature variability” is defined complicates direct comparisons across studies.^25^, ^26^, ^27^

Importantly, Ni et al. recently reported in a nationwide Swedish time-stratified case-crossover study that upward temperature shifts were associated with increased MI hospital admissions at lag 0.^28^ Our findings align directionally with this evidence and extend it in clinically relevant ways by demonstrating associations of uTS with a broader spectrum of endpoints in patients with established ASCVD, including ACS, stroke, heart failure hospitalizations, and all-cause mortality, as well as by describing outcome-specific lag patterns and translating relative risks into absolute burden.

From a mechanistic perspective, sudden temperature increases may challenge thermoregulation and cardiovascular homeostasis, including autonomic changes and volume stress related to peripheral vasodilation and dehydration.^29^, ^30^, ^31^ In addition, inflammatory pathways have been linked to temperature variability, with prior work reporting higher hs-CRP levels with greater variability and stronger susceptibility among older adults.^32^ Consistent with this vulnerability framework, we observed that associations were largely driven by older individuals aged 65–85 years, for whom impaired thermoregulation may increase susceptibility.^33^ We also observed outcome-specific temporal patterns (e.g., acute effects for some endpoints and more delayed peaks for others), which may help frame clinically relevant risk windows for prevention and preparedness.

Several inherent limitations warrant consideration. First, the study was restricted to individuals with ASCVD in Bavaria, Germany, and findings may not generalize to other healthcare settings or to regions with substantially different climates. While this may limit generalizability, the large sample and high-resolution exposure assignment support the internal validity of the observed associations in this vulnerable population. Second, exposures were assigned using outdoor gridded estimates rather than indoor temperature exposure. This likely introduces non-differential exposure measurement error, which would be expected to attenuate associations toward the null. Third, since claims data are collected for administrative purposes, outcome misclassification is possible. Fourth, the absence of CVD specific mortality data and the use of all-cause mortality may limit interpretability. To improve specificity, outcomes were defined using inpatient claims, and ACS/AMI definitions incorporated diagnosis codes together with relevant coronary procedure codes within a prespecified time window. The employed validation algorithms demonstrated high positive predicted values and comparable outcome rates in randomized trials.^13^

### Conclusion

Our findings highlight temperature variability as an independent short term environmental risk factor in patients with ASCVD and support preparedness for acute risk during abrupt temperature shifts, without asserting that our study quantifies effects of climate change.

## Contributors

Katharina Lechner: Conceptualization, Data curation, Formal analysis, Funding acquisition, Investigation, Methodology, Project administration, Resources, Validation, Writing–original draft, Writing–review & editing, responsible for the decision to submit the manuscript. Siqi Zhang: Data curation, Formal analysis, Investigation, Methodology, Validation, Visualization, Writing–original draft, Writing–review & editing, responsible for the decision to submit the manuscript. Nils Krüger: Conceptualization, Data curation, Formal analysis, Investigation, Methodology, Validation, Writing–original draft, Writing–review & editing, responsible for the decision to submit the manuscript. Johannes Krefting: Data curation, Formal analysis, Writing–review & editing. Kathrin Wolf: Data curation, Formal analysis, Visualization, Writing–review & editing. Marco Dallavalle: Data curation, Formal analysis, Visualization, Writing–review & editing. Tobias Dreischulte: Writing–review & editing. Frank Offenborn: Data curation, Formal analysis, Writing–review & editing. Fabian Starnecker: Writing–review & editing. Moritz von Scheidt: Data curation, Writing–review & editing. Kai Chen: Supervision, Validation, Writing–review & editing. Annette Peters: Supervision, Validation, Writing–review & editing. Susanne Breitner: Conceptualization, Data curation, Formal analysis, Investigation, Methodology, Resources, Supervision, Validation, Writing–review & editing, responsible for the decision to submit the manuscript. Alexandra Schneider: Conceptualization, Data curation, Formal analysis, Investigation, Methodology, Resources, Supervision, Validation, Writing–review & editing, responsible for the decision to submit the manuscript. Heribert Schunkert: Conceptualization, Data curation, Formal analysis, Investigation, Methodology, Project administration, Resources, Supervision, Validation, Visualization, Writing–review & editing, responsible for the decision to submit the manuscript.

All shared first and senior authors were responsible for the decision to submit the manuscript. Siqi Zhang, Nils Krüger and Susanne Breitner have directly accessed and verified the underlying data.

## Data sharing statement

The data underlying this study are available from the corresponding author upon reasonable request. Data sharing may be subject to ethical approval, institutional requirements, and applicable data protection regulations. No accession code applies.

## Declaration of interests

Moritz von Scheidt reports support for the present manuscript from the Bavarian Government through DigiMed Bayern, for which he serves as Co-PI. He has received grants or contracts from HORIZON/EU funding for EuroHeartPath, a HORIZON-JU-IHI grant for which he serves as Co-PI; HORIZON EIC funding for MIRACLE, for which he is a member; the Corona Foundation for a Junior Research Group, for which he serves as PI and which is ongoing from 2021 to 2026; BMBF/DZHK funding for multiple grants, for which he serves as Co-PI or member; the DZG Innovation Fund for MetaboCHIP, for which he serves as Co-PI and which is ongoing from 2025 to 2027; and Fondation Leducq funding for PlaqOmics, for which he is a member and which is ongoing from 2021 to 2025. He also reports an unpaid leadership role as initiator and head of the German CHIP Registry e.V., a non-profit organization.

Relevant to this manuscript, Katharina Lechner reports support for the present manuscript from the German Heart Foundation, grant no. F/58/21. The German Heart Foundation grant amounted to 67,556 Euro. Katharina Lechner also reports an unpaid leadership or fiduciary role through a mandate for the S2k guideline ‘Heat-related health disorders’ of the German Society of General Practice and Family Medicine.

Heribert Schunkert reports personal fees during 2023–2026 from AMGEN, APONTIS, Astra-Zeneca, Bayer Vital GmbH, Boehringer Ingelheim, Bristol-Myers Squibb, Daiichi Sankyo, MSD SHARP & DOHME, Novartis, NovoNordisk, Pharmacosmos, Sanofi Aventis, Servier, and Synlab. These included fees for lectures from AMGEN, APONTIS, Astra-Zeneca, Bayer Vital GmbH, Bristol-Myers Squibb, Daiichi Sankyo, MSD SHARP & DOHME, Novartis, NovoNordisk, Pharmacosmos, Sanofi Aventis, and Synlab, and advisory board or consultancy activities for Boehringer Ingelheim, Daiichi Sankyo, MSD SHARP & DOHME, Servier, and Synlab.
