# Supplementary material for: Upward Air Temperature Shifts and Acute Cardiovascular Events in Individuals with Atherosclerotic Cardiovascular Disease: A Time-Stratified Case-Crossover Study
Source: Lancet Reg Health Eur. 2026 Jun 4;67:101730. doi: 10.1016/j.lanepe.2026.101730 (PMC13262291; doi:10.1016/j.lanepe.2026.101730)
Supplement: Supplementary Figures and Tables [file mmc1.docx]

Table S1. Definitions of comorbidities and medications

| **Characteristic** | **ICD/ATC Codes** |
| --- | --- |
| **Comorbidities** |  |
| Chronic ischemic heart disease | I25 |
| Peripheral artery disease | I70.2 |
| Carotid stenosis | I65.2 |
| Acute stroke | I63, I64 |
| Chronic heart failure | I50 |
| Chronic kidney disease | N18 |
| Arterial hypertension | I10, I15 |
| Diabetes mellitus | E10, E11, E12, E13, E14 |
| Dyslipidemia | E78 |
| Adipositas | E66 |
| Nicotine abuse | F17 |
| **Medications** |  |
| Antiplatelet agents | B01AC |
| ACE inhibitors | C09A, C09BA01, C09BA02, C09BA03, C09BA04, C09BA05, C09BA06, C09BA07, C09BA08, C09BA09, C09BA12, C09BA13, C09BA15, C09BA21, C09BA22, C09BA23, C09BA25, C09BA26, C09BA27, C09BA28, C09BA29, C09BA33, C09BA35, C09BA54, C09BA55, C09BB |
| Angiotensin II receptor blockers | C09CA, C09DA01, C09DA02, C09DA03, C09DA04, C09DA06, C09DA07, C09DA08, C09DA09, C09DA10, C09DA21, C09DA22, C09DA23, C09DA24, C09DA26, C09DA27, C09DA28, C09DB |
| Calcium channel blockers | C07FB, C08, C09BB, C09DB |
| Beta blocking agents | C07A, C07B, C07C, C07D, C07E, C07FB |
| Diuretics | C03A, C03EA, C07B, C07D, C09BA21, C09BA22, C09BA23, C09BA25, C09BA26, C09BA27, C09BA28, C09BA29, C09BA33, C09BA35, C09BA54, C09DA21, C09DA22, C09DA23, C09DA24, C09DA26, C09DA27, C09DA28, C03B, C03DB, C03EA, C03EB, C03EC, C03X, C09BA01, C09BA02, C09BA03, C09BA04, C09BA05, C09BA06, C09BA07, C09BA08, C09BA09, C09BA12, C09BA13, C09BA15, C09DA01, C09DA02, C09DA03, C09DA04, C09DA06, C09DA07, C09DA08, C09DA09, C09DA10, C03C, C03EB, C03ED, C07C, C07D, C09BA55 |
| Aldosteron antagonists | C03DA, C03EC, C03ED |
| Insulins and analogues | A10A |
| Other antidiabetic agents | A10B |

Table S2. Population attributable risk (PAR) for cause-specific hospital admissions and deaths is the estimated proportion (%) of events in the population that would be prevented if the exposure were reduced to the reference level (assuming a causal relationship). Values shown as PAR% (95% CI), where reported.

| Outcome | Number (/100,000 participants per year) | 95% CI_lower | 95% CI_upper |
| --- | --- | --- | --- |
| AMI | 5 | 3 | 7 |
| ACS | 7 | 5 | 10 |
| HF | 10 | 4 | 16 |
| Stroke | 6 | 3 | 9 |
| Mortality | 23 | 18 | 29 |
| MACE | 26 | 21 | 31 |

Table S3. Pooled relative risks (with 95% confidence interval) for first hospital admissions due to acute coronary syndrome, acute myocardial infarction, heart failure, and stroke, as well as for all-cause mortality and first major adverse cardiovascular events associated with per 5°C greater upward temperature shift in different subgroups at lag 0 day.

| Analysis | ACS | AMI | HF | Stroke | Mortality | MACE |
| --- | --- | --- | --- | --- | --- | --- |
| Age<65yrs | 1.02 (1.01, 1.03)^*^ | 1.03 (1.00, 1.06)^*^ | 1.01 (0.99, 1.04) | 1.03 (1.00, 1.05)^*^ | 1.01 (0.99, 1.04) | 1.02 (1.01, 1.04)^*^ |
| Age 65-85yrs | 1.04 (1.02, 1.05)^*^ | 1.03 (1.01, 1.06)^*^ | 1.01 (1.00, 1.02)^*^ | 1.02 (1.01, 1.04)^*^ | 1.04 (1.03, 1.04)^*^ | 1.03 (1.02, 1.04)^*^ |
| Age>85yrs | 1.02 (0.94, 1.10) | 0.99 (0.90, 1.09) | 1.02 (1.00, 1.03)^*^ | 1.00 (0.99, 1.02) | 1.03 (1.02, 1.04)^*^ | 1.02 (1.01, 1.03)^*^ |
| Males | 1.03 (1.02, 1.04)^*^ | 1.03 (1.01, 1.05)^*^ | 1.01 (1.00, 1.02)^*^ | 1.03 (1.01, 1.04)^*^ | 1.03 (1.02, 1.04)^*^ | 1.03 (1.02, 1.04)^*^ |
| Females | 1.02 (1.00, 1.04)^*^ | 1.01 (0.99, 1.04) | 1.01 (1.00, 1.02)^*^ | 1.01 (1.00, 1.03)^*^ | 1.03 (1.02, 1.04)^*^ | 1.02 (1.01, 1.03)^*^ |
| Warm season^a^ | 1.03 (1.01, 1.05)^*^ | 1.03 (1.01, 1.05)^*^ | 1.01 (1.00, 1.02) | 1.0172 (0.1, 1.03) | 1.05 (1.04, 1.06)^*^ | 1.03 (1.02, 1.04)^*^ |
| Cold season^a^ | 1.01 (0.10, 1.02) | 1.00 (0.98, 1.02) | 1.01 (0.10, 1.02) | 1.01 (0.10, 1.03) | 1.02 (1.00, 1.03)^*^ | 1.01 (1.00, 1.02)^*^ |

Conditional Poisson regression with a linear term of temperature variability was conducted for each postcode area. Postcode-specific estimates were pooled by random-effects meta-analysis.

^*^p<0.05.

ACS=acute coronary syndrome, AMI=acute myocardial infarction, HF=heart failure, MACE=major adverse cardiovascular events.

Table S4. Pooled relative risks (with 95% confidence interval) for first hospital admissions due to acute coronary syndrome, acute myocardial infarction, heart failure, and stroke, as well as for all-cause mortality and first major adverse cardiovascular events associated with per 5°C greater upward temperature shift with adjustment for other exposure metrics at lag 0 day.

| Adjustment | ACS | AMI | HF | Stroke | Mortality | MACE |
| --- | --- | --- | --- | --- | --- | --- |
| Main | 1.03 (1.02, 1.0384) | 1.03 (1.02, 1.04) | 1.01 (1.01, 1.02) | 1.02 (1.01, 1.03) | 1.03 (1.02, 1.04) | 1.03 (1.02, 1.03) |
| Daily tmean | 1.04 (1.03, 1.06) | 1.04 (1.02, 1.06) | 1.02 (1.01, 1.03) | 1.03 (1.01, 1.05) | 1.03 (1.02, 1.04) | 1.03 (1.02, 1.04) |
| Preceding 7-day tmean | 1.02 (1.02, 1.03) | 1.02 (1.01, 1.04) | 1.01 (1.01, 1.02) | 1.02 (1.01, 1.03) | 1.03 (1.03, 1.04) | 1.03 (1.02, 1.03) |
| RH | 1.04 (1.02, 1.05) | 1.03 (1.02, 1.04) | 1.02 (1.01, 1.03) | 1.02 (1.01, 1.03) | 1.03 (1.03, 1.04) | 1.03 (1.02, 1.03) |
| PM_10_ | 1.03 (1.02, 1.04) | 1.03 (1.02, 1.04) | 1.01 (1.01, 1.02) | 1.02 (1.01, 1.03) | 1.03 (1.02, 1.04) | 1.03 (1.02, 1.03) |
| PM_2.5_ | 1.03 (1.02, 1.04) | 1.03 (1.02, 1.04) | 1.01 (1.01, 1.02) | 1.02 (1.01, 1.03) | 1.03 (1.02, 1.04) | 1.03 (1.02, 1.03) |
| NO_2_ | 1.03 (1.02, 1.04) | 1.03 (1.02, 1.04) | 1.01 (1.01, 1.02) | 1.02 (1.01, 1.0293) | 1.03 (1.02, 1.04) | 1.03 (1.02, 1.03) |
| O_3_ | 1.04 (1.03, 1.05) | 1.03 (1.02, 1.05) | 1.02 (1.01, 1.03) | 1.02 (1.01, 1.03) | 1.03 (1.02, 1.04) | 1.03 (1.02, 1.03) |

Conditional Poisson regression with a linear term of temperature variability was used. Present-day daily mean temperature, preceding 7-day average temperature, and present-day RH were adjusted for as a natural spline; present-day air pollutants were adjusted for as a linear term.

ACS=acute coronary syndrome, AMI=acute myocardial infarction, HF=heart failure, MACE=major adverse cardiovascular events, NO_2_=nitrogen dioxide, O_3_=ozone, PM_2.5_= particulate matter with an aerodynamic diameter of ≤ 2.5 µm, PM_10_=particulate matter with an aerodynamic diameter of ≤ 10 µm, RH=relative humidity.

Table S5. Pearson correlation coefficients between area-specific daily exposure metrics.

|  | Temperature | TS | RH | PM_10_ | PM_2.5_ | NO_2_ |
| --- | --- | --- | --- | --- | --- | --- |
| Temperature | 1.00 |  |  |  |  |  |
| TS | 0.32 | 1.00 |  |  |  |  |
| RH | -0.55 | -0.29 | 1.00 |  |  |  |
| PM_10_ | -0.17 | 0.09 | -0.05 | 1.00 |  |  |
| PM_2.5_ | -0.29 | 0.04 | 0.06 | 0.91 | 1.00 |  |
| NO_2_ | -0.37 | -0.01 | 0.19 | 0.52 | 0.48 | 1.00 |
| O_3_ | 0.57 | 0.20 | -0.69 | -0.21 | -0.25 | -0.55 |


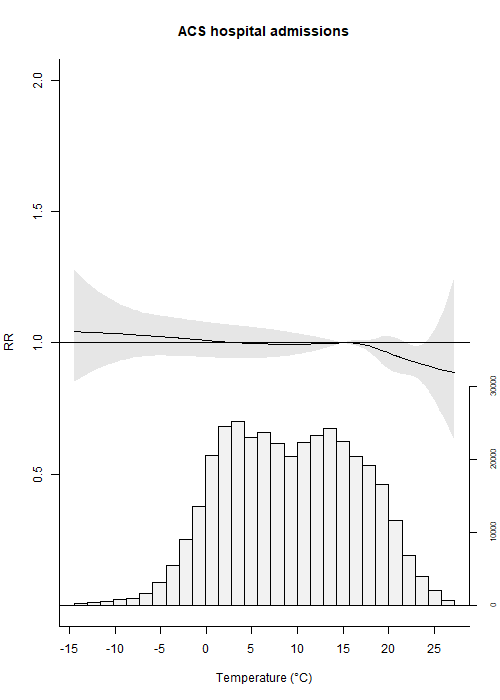

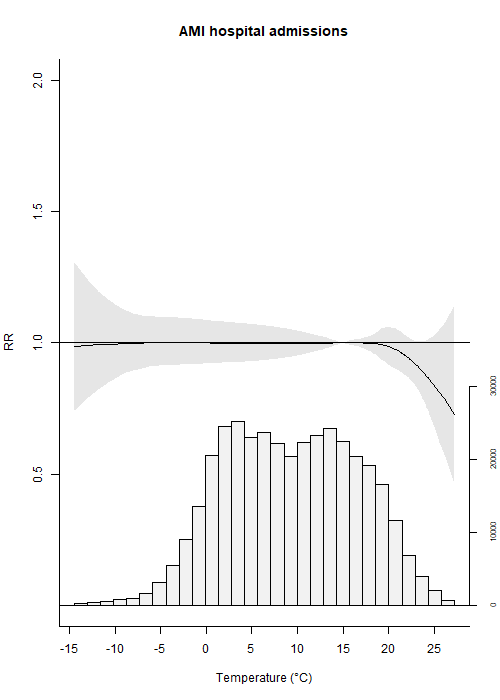

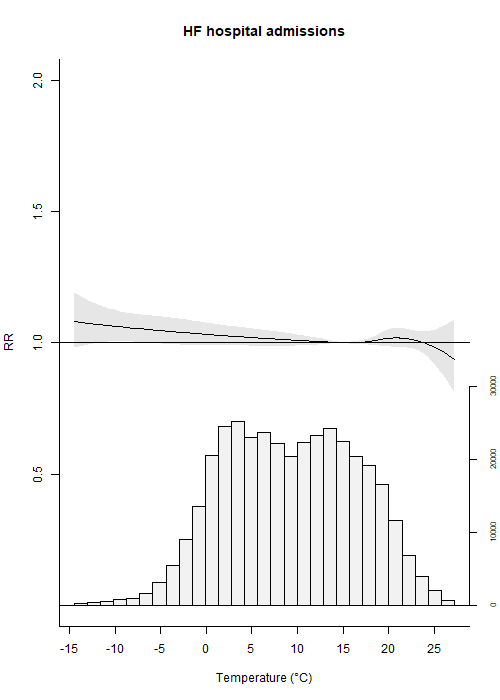

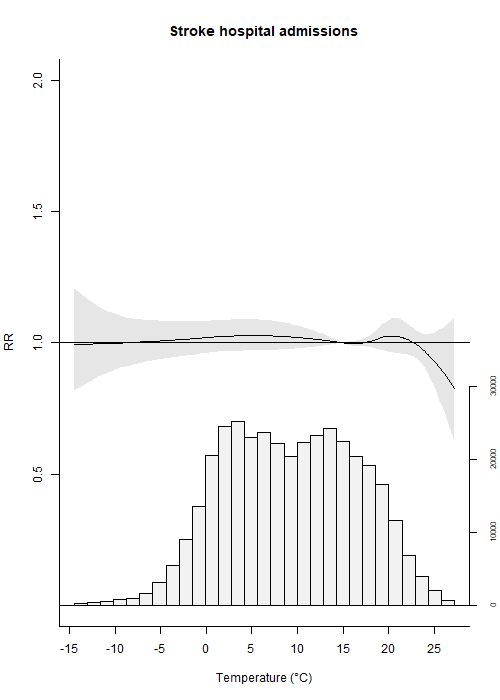

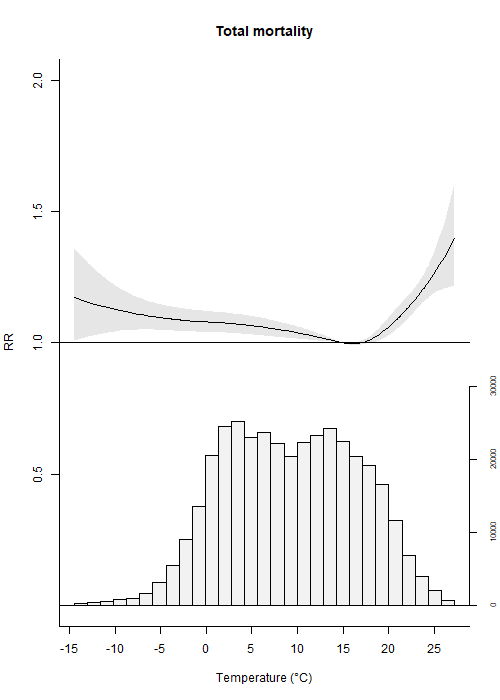

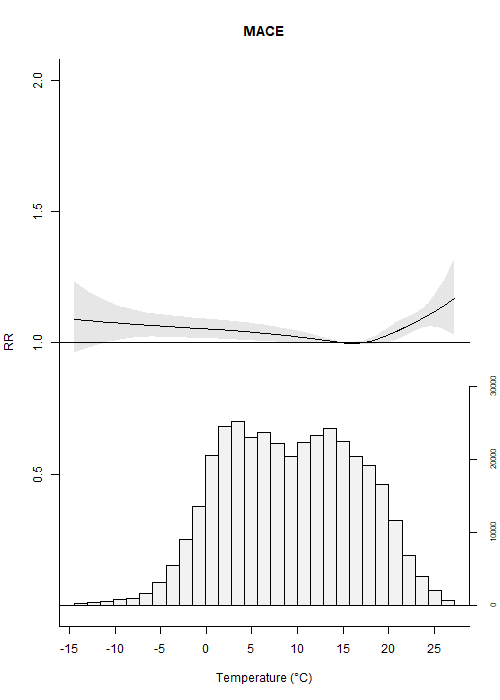


Figure S1. Exposure-response relationships between daily mean temperature and first hospital admissions due to acute coronary syndrome, myocardial infarction, heart failure, and stroke, all-cause mortality, and major adverse cardiovascular events using lag 0-6 days.


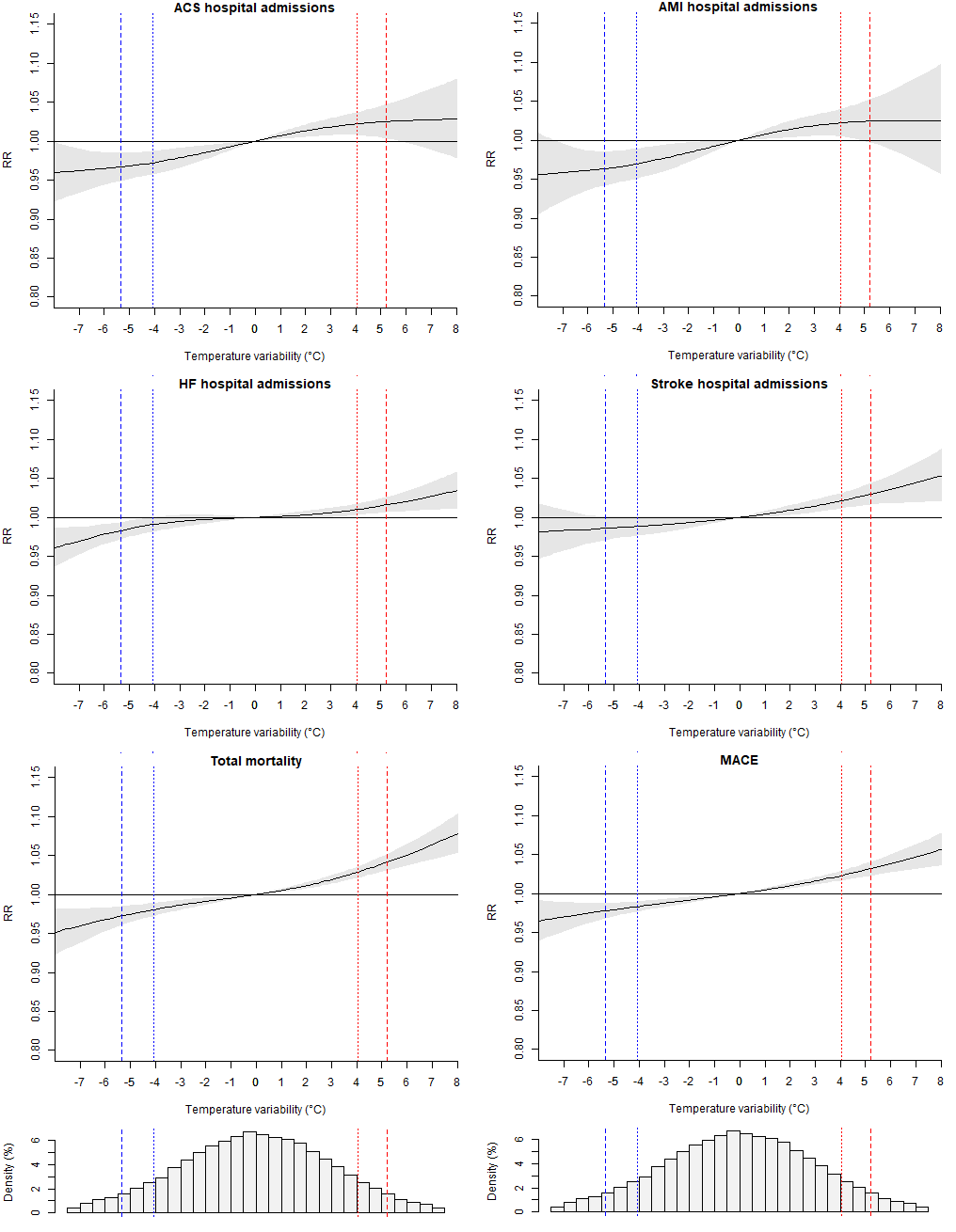


Figure S2. Exposure-response relationships between daily mean temperature variability and first hospital admissions due to acute coronary syndrome, acute myocardial infarction, heart failure, and stroke, all-cause mortality, and major adverse cardiovascular events at lag 0 day.

The exposure-response relationships were estimated using conditional Poisson regressions with distributed lag non-linear models. Present-day temperature variability was included in the regressions as a natural spline. To avoid the influence of extreme temperature variability values, the outliers in temperature variability lower than the 1st percentile or higher than the 99th percentile were excluded from the regressions. The histograms show the distribution of the temperature variability. The blue and red dashed lines represent the 5th and 95th percentiles of temperature variability distribution, respectively. The blue and red dotted lines represent the 10th and 90th percentiles of the temperature variability distribution, respectively. ACS=acute coronary syndrome, AMI=acute myocardial infarction, HF=heart failure, MACE=major adverse cardiovascular events.
